# Patients with Mild Traumatic Brain Injury Recruited from Both Hospital and Primary Care Settings: A Controlled Longitudinal Magnetic Resonance Imaging Study

**DOI:** 10.1089/neu.2018.6360

**Published:** 2019-10-23

**Authors:** Cathrine Elisabeth Einarsen, Kent Gøran Moen, Asta Kristine Håberg, Live Eikenes, Kjell Arne Kvistad, Jian Xu, Hans Kristian Moe, Marie Hexeberg Tollefsen, Anne Vik, Toril Skandsen

**Affiliations:** ^1^ Department of Neuromedicine and Movement Science, Norwegian University of Science and Technology, Trondheim, Norway.; ^2^ Department of Physical Medicine and Rehabilitation, St. Olavs Hospital, Trondheim University Hospital, Trondheim, Norway.; ^3^ Department of Radiology, Levanger Hospital, Nord-Trøndelag Hospital Trust, Levanger, Norway.; ^4^ Department of Radiology and Nuclear Medicine, St. Olavs Hospital, Trondheim University Hospital, Trondheim, Norway.; ^5^ Department of Circulation and Medical Imaging Faculty of Medicine and Health Sciences, Norwegian University of Science and Technology, Trondheim, Norway.; ^6^ Department of Neurosurgery, St. Olavs Hospital, Trondheim University Hospital, Trondheim, Norway.

**Keywords:** diffuse axonal injury, MRI, PCS, prospective study, white matter hyperintensities

## Abstract

With an emphasis on traumatic axonal injury (TAI), frequency and evolution of traumatic intracranial lesions on 3T clinical magnetic resonance imaging (MRI) were assessed in a combined hospital and community-based study of patients with mild traumatic brain injury (mTBI). The findings were related to post-concussion symptoms (PCS) at 3 and 12 months. Prospectively, 194 patients (16–60 years of age) were recruited from the emergency departments at a level 1 trauma center and a municipal outpatient clinic into the Trondheim mTBI follow-up study. MRI was acquired within 72 h (*n* = 194) and at 3 (*n* = 165) and 12 months (*n* = 152) in patients and community controls (*n* = 78). The protocol included T2, diffusion weighted imaging, fluid attenuated inversion recovery (FLAIR), and susceptibility weighted imaging (SWI). PCS was assessed with British Columbia Post Concussion Symptom Inventory in patients and controls. Traumatic lesions were present in 12% on very early MRI, and in 5% when computed tomography (CT) was negative. TAI was found in 6% and persisted for 12 months on SWI, whereas TAI lesions on FLAIR disappeared or became less conspicuous on follow-up. PCS occurred in 33% of patients with lesions on MRI and in 19% in patients without lesions at 3 months (*p* = 0.12) and in 21% with lesions and 14% without lesions at 12 months (*p* = 0.49). Very early MRI depicted cases of TAI in patients with mTBI with microbleeds persisting for 12 months. Patients with traumatic lesions may have a more protracted recovery, but the study was underpowered to detect significant differences for PCS because of the low frequency of trauma-related MRI lesions.

## Introduction

Despite extensive research into novel imaging biomarkers of mild traumatic brain injury (mTBI),^1,2^ such methods are not ready for diagnostic use in the individual patient. Therefore, presence of visible TBI related lesions on computed tomography (CT) and clinical magnetic resonance imaging (MRI) reported by neuroradiologists is still the standard option in clinical practice. The reported frequency of traumatic lesions on CT and MRI in mTBI varies enormously across studies, from 5% to 39% for CT^3–7^ and from 6% to 75% for MRI.^8–11^ MRI is more sensitive than CT, primarily linked to lack of detection of traumatic axonal injury (TAI) on CT.^12^ However, the significance of detecting TAI lesions in mTBI is unclear, and there are no guidelines regarding which patients that should be referred to a clinical MRI examination after mTBI. Even less is known regarding the optimal timing of an MRI examination, because the evolution of lesions over time never has been studied longitudinally in a cohort of mTBI: Such information is needed to clarify if there is a time window of opportunity to uncover traumatic lesions on MRI, which should be of both clinical and medicolegal interest. Another unresolved issue is the long-term consequence of traumatic MRI lesions on outcome such as post-concussion symptoms (PCS) in mTBI. Both worse outcome^10,13^ and no effect on outcome of TBI findings on MRI have been reported.^3,8,9,14–17^ The heterogeneity and inconsistency in findings of previous studies, regarding both the frequency and long-term consequences of MRI findings can probably be explained by a huge variation in patient selection, scan protocols, magnetic field strength, timing of examinations, and methods for outcome assessment. Another issue in mTBI research is the large group of patients treated outside hospitals. This group is likely under-represented in studies of mTBI,^18^ leading to a biased sample with more severe mTBI patients being included.

Here we present results from longitudinal MRI examinations in the population-based Trondheim mTBI follow-up study using a state-of-the-art scan protocol.^19^ Patients with mTBI were enrolled prospectively, from both the hospital and primary care setting, using few exclusion criteria. Further, the patients in this MRI study were compared with eligible patients with mTBI who received a basic follow-up without MRI.

The first aim of the study was to examine the frequency and evolution over time of traumatic intracranial lesions on clinical MRI performed within 72 h and at 3 and 12 months after an mTBI, with a particular focus of TAI lesions. A community control group was also scanned, and presence of microbleeds and non-traumatic white matter hyperintensities (WMH) were compared between the patients with mTBI and the controls. The second aim was to examine the association between traumatic lesions on MRI within 72 h and the presence of PCS in patients and controls.

## Methods

### Patients in the Trondheim mTBI follow-up study

The inclusion period in the prospective cohort study Trondheim mTBI follow-up study was April 2014 to December 2015. Patients were recruited from two emergency departments (EDs): at St. Olavs Hospital (Trondheim University Hospital), a regional level 1 trauma center in Trondheim, Norway, and Trondheim Municipal Emergency clinic, a general practitioner-run, outpatient clinic. In the study period, the two EDs were located in the same building and used the same CT service. Patients could be referred from the municipal ED to the trauma center for hospitalization as clinically indicated; that is, in cases with CT findings.

Inclusion criteria were: (1) having sustained an mTBI according to the World Health Organization criteria (a) Glasgow Coma Scale (GCS) score 13–15 and (b) either loss of consciousness (LOC) <30 min, confusion, or post-traumatic amnesia (PTA) <24 h^20^ and (2) age between 16 and 60 years. Exclusion criteria were: (1) non-fluency in the Norwegian language; (2) having a pre-existing severe neurological, psychiatric, somatic, or substance use determined to be severe enough to likely interfere with follow-up and outcome assessment; (3) having a prior history of a complicated mild (prior traumatic lesions on CT), moderate, or severe TBI; (4) other major trauma; or (5) presentation >48 h after the initial trauma.

The patients included in the Trondheim mTBI follow-up study have been shown to be largely representative of patients with mTBI as described in detail elsewhere.^19^

### Patients in the MRI study and their matched control subjects

**FIG. 1.**
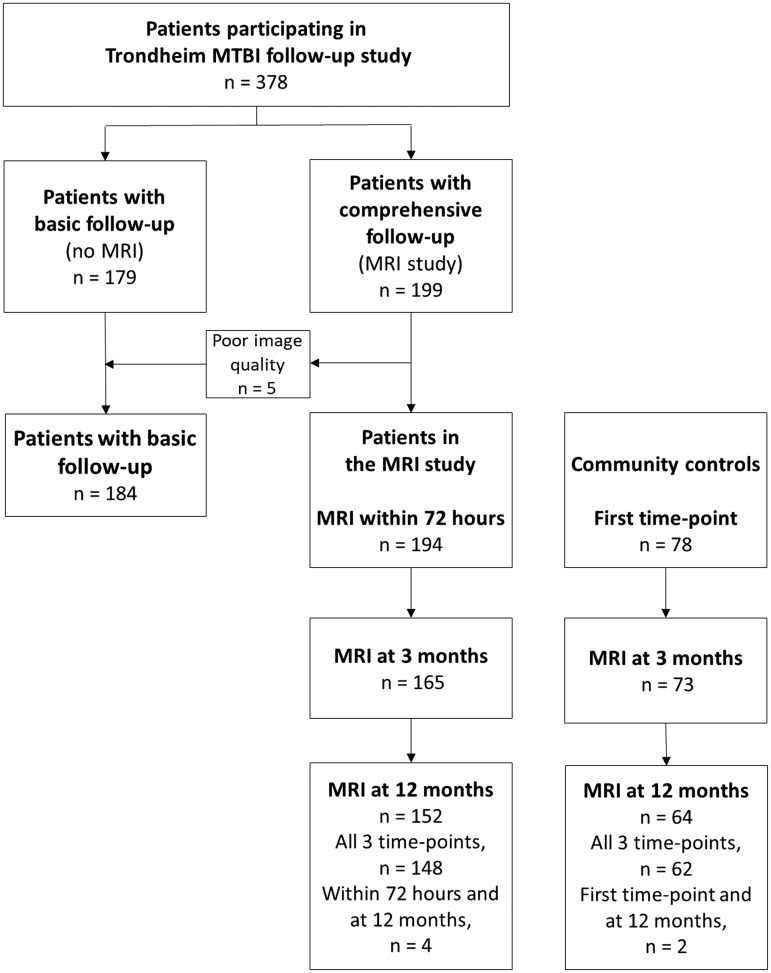
Flow chart of patients included in the Trondheim mild traumatic brain injury (mTBI) follow-up study. Magnetic resonance imaging (MRI) was performed at all time points in 148 patients.

In total, 378 patients were enrolled in the Trondheim mTBI follow-up study (Fig. 1). Of these, 194 patients participated in a comprehensive data collection (MRI study) if they consented to MRI, had no MRI contraindications, MRI scanning could be performed very early, and they lived within a 1 h drive from the study hospital. MRI scans were acquired within 72 h (very early), and at 3 and 12 months post-injury. The remaining patients (*n* = 184) received a basic follow-up with telephone interviews and questionnaires (basic follow-up).

A convenience sample of community controls (*n* = 78) was recruited, matched at the group level, to the participants in the MRI study for age, sex, and education. Exclusion criteria were the same as for the mTBI group, except that they could not receive treatment for severe psychiatric conditions, even if they might be able to comply with follow-up. The latter criterion was slightly different for patients and controls, because our goal was to establish an mTBI cohort as representative as possible, and a control group with reasonably good brain health. The controls underwent the same follow-up as the patients in the MRI study, including MRI at inclusion at 3 and 12 months, and outcome evaluation at 3 and 12 months.

### Clinical variables and outcome

Clinical variables and outcome data were collected from patient interviews and medical records. Previous mTBI was defined as one or more brain injuries fulfilling the same diagnostic criteria as applied for the current injury. The GCS score was assessed by study personnel or obtained from the patient's medical records. If lacking, the history and clinical descriptions in the medical record were used to estimate a score. LOC was registered as yes or no if witnessed, otherwise it was as unknown. Duration of PTA was recorded as the time after injury for which the patient had no continuous memory, and dichotomized into <1 h and 1–24 h.

Outcome was assessed by telephone at 3 and 12 months 1–2 weeks before the patients and controls came for the MRI examination, and the interviewer was blinded to clinical information. Outcome was measured with the British Columbia Post Concussion Symptom Inventory (BC-PSI),^21^ which is a symptom inventory based on the 10th Revision of the International Statistical Classification of Diseases and Related Health Problems (ICD-10) symptom criteria for PCS.^22^ The patients and controls were asked to rate the frequency (0 = not at all, 5 = constantly) and intensity (0 = not at all, 5 = very severe problem) of 13 symptoms: (1) headaches, (2) dizziness, (3) nausea, (4) fatigue, (5) sensitivity to noises, (6) irritability, (7) sadness, (8) feeling nervous or tense, (9) temper problems, (10) poor concentration, (11) memory problems, (12) difficulty reading, and (13) poor sleep. Additionally, three co-occurring life problems were registered: (1) effects of alcohol consumption, (2) worrying/ dwelling on symptoms, and (3) self-perception of brain damage. Participants were classified as having PCS if their symptoms scored on the BC-PSI items as being moderate or greater on three or more of six ICD-10 Category C criteria.^23^

### Head CT

Non-contrast CT (*n* = 162) was performed on a Siemens Somatom Sensation 64 row scanner as part of the initial clinical assessment, according to the Scandinavian Guidelines for Head Injury Management.^24^

CT readings were first obtained from the radiology report. The intracranial traumatic findings on CT were classified into: (1) contusion, (2) epidural hematoma (EDH), (3) traumatic subarachnoid hemorrhage (tSAH), and (4) subdural hematoma (SDH). The CT scans from patients with intracranial traumatic findings on MRI were later reviewed by an experienced neuroradiologist (K.A.K.) and a consultant in physical medicine and rehabilitation (C.E.E.). Two additional findings were thereby identified, one tSAH and one SDH.

### Brain MRI

MRI was performed on a 3.0T Siemens Skyra system (Siemens Healthcare, Erlangen, Germany), software version E11C, with a 32 channel head coil. The image protocol consisted of a series of clinical MRI sequences: (1) three dimensional (3D) T1-weighted magnetization-prepared rapid acquisition with gradient echo (MPRAGE) (repetition time [TR]: 2300 ms, echo time [TE] 4.21 ms, slice thickness 1.0, voxel size: 1.0 × 1.0 × 1.0 mm^3^); (2) two dimensional (2D) diffusion weighted imaging (DWI) (TR: 6800 ms, TE 91 ms, slice thickness 4.0, voxel size: 1.2 × 1.2 × 4.0 mm^3^); (3) 3D T2 space (TR: 3200 ms, TE 371 ms, slice thickness 0.9, voxel size: 0.4 × 0.4 × 0.9 mm^3^); (4) 3D T2-weighted fluid attenuated inversion recovery (FLAIR): (TR: 5000 ms, TE 389 ms, slice thickness 1.0, flip angle: T2var, voxel size: 1.0 × 1.0 × 1.0 mm^3^); and (5) 3D T2-weighted susceptibility weighted imaging (SWI) (TR: 27 ms, TE 20 ms, slice thickness 1.5, voxel size: 0.9 × 0.9 × 1.5 mm^3^).

The MRIs from all three time points were read and reported by K.A.K. and a resident in radiology (J.X.). The intracranial traumatic MRI findings were categorized into: (1) TAI, (2) contusion, (3) EDH, (4) SDH, and (5) tSAH.

The SWI scans were inspected for hypointense foci or microbleeds.^25^ The microbleeds were classified as TAI lesions when they had the appearance of small, rounded, or circular, well-defined hypointense lesions with clear margins, often in clusters, located in the typical locations (the lobar white matter, corpus callosum, brainstem, basal ganglia, or thalamus), and not in the gyrus-sulcus pattern.^26^ Superficial microbleeds in the cortex were defined as contusions.

The FLAIR and DWI scans were inspected for hyperintense lesions, and the lesions were classified as TAI lesions if they occurred in the typical locations for TAI, and as contusions if they were located in the cortex. The early edema observed on acute (< 72 h) FLAIR and DWI resolved with time. Depending on the resulting type of tissue injury, in the chronic phase, the signal can remain elevated in presence of gliosis, reduced in cases of necrosis, or appear similar to normal tissue, albeit its microstructure may be altered.^26,27^

**FIG. 2.**
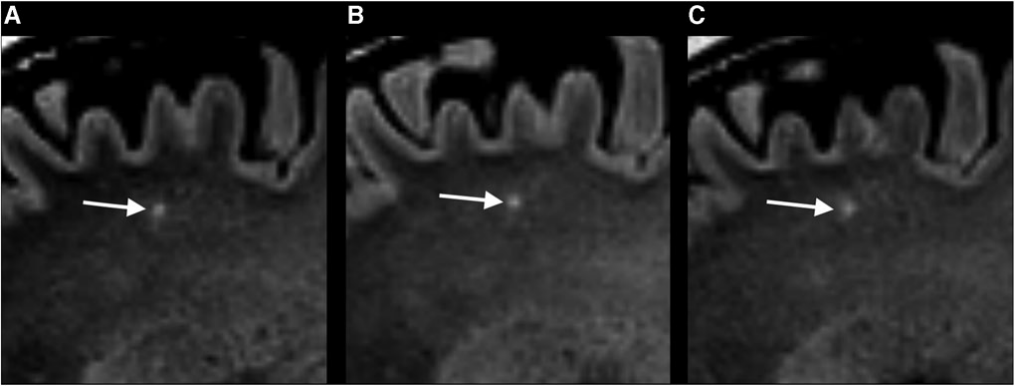
**(A–C)** A non-traumatic white-matter hyperintensity (WMH) on fluid attenuated inversion recovery (FLAIR) scans in the sagittal plane of the same patient at three different time points. **(A)** The very early FLAIR scan showed a hyperintense lesion in the deep gray matter in the left frontal lobe (arrow) that persisted at the **(B)** 3 month and **(C)** 12 month examination. The lesion was not present at the susceptibility weighted image scan at any time point.

An additional evaluation of the FLAIR and the SWI scans of all participants at all time-points was performed by C.E.E. supervised by K.A.K., to assess the presence of microbleeds or non-traumatic WMH in the patients with mTBI and the controls. A WMH was classified as non-traumatic if present in white matter on FLAIR, if no hypointense lesion could be observed in the same region on SWI, and, when there were follow-up scans, if the WMH retained its contrast, size, and shape across MRI time points (Fig. 2). Participants were classified having WMH if one or more WMH was found. The lesions were not described with relation to size and location, and no attempt was made to distinguish non-traumatic WMH into unspecific versus age related.

### Inter-rater reliability (IRR)

A resident in radiology (K.G.M.), blinded to clinical information and results of previous readings, read the very early MRIs of all patients with intracranial findings (detected in the first step of the reading, *n* = 22) and of an approximately threefold higher number of patients without intracranial findings (*n* = 60). The scans were presented in a random order. K.G.M.'s findings were compared with the consensus-based findings reported by C.E.E., J.X., and K.A.K. to examine the IRR of the method applied to the clinical MRI reading.

### Statistical analysis

Patient and injury characteristics are presented as percentages, means with standard deviation (SD), or median with interquartile range (IQR) (25th to 75th percentile). Between-group differences were analyzed with the Mann–Whitney *U* test because of non-normal distribution. The χ^2^ test was used for comparison of proportions, and the exact unconditional test was used when the expected cell values were <5.^28^ The association between MRI findings and PCS was explored with binary logistical regression and presented as odds ratio (OR) with 95% confidence interval (CI), where “non-traumatic MRI lesions” was the reference category. IRR was evaluated with Cohen's κ statistics for categorical variables. A κ value <0.20 was poor, a value of 0.21–0.40 was fair, a value of 0.41–0.60 was moderate, a value of 0.61–0.80 was good and a value >0.80 was very good.^29^

A *p* value of <0.05 was considered statistically significant for all analysis. IBM Statistical Package for the Social Sciences (SPSS^©^) Statistics version 25 was used for all analysis.

### Ethics

The study was approved by the regional committee for research ethics (REK 2013/754) and was conducted in accordance with the Helsinki declaration. All participants, and a caregiver of participants <18 years of age, gave informed consent. Participants received a gift card worth 40 or 60 USD, depending on the length of the session, as compensation for costs.

## Results

Out of the 378 patients included in the Trondheim mTBI follow-up study, 194 (51%) participated in the MRI study (Fig. 1). The median age was higher in the MRI study than in the basic follow-up (27 years vs. 24 years, *p* = 0.008) (Table 1). In the MRI study, 39% were injured by a fall, 81% had GCS score 15, 71% had observed LOC, and 30 % had PTA 1–24 h. Previous uncomplicated mTBI was more frequent among patients in the MRI study than in the controls (24% vs. 9%, *p* = 0.004), otherwise no differences were uncovered (Table 2).

**Table 1.** Patients Characteristics: MRI Study and Basic Follow-up

*Variable*	*All* n* = 378*	*MRI study* n* =* 194 (51%)	*Basic follow-up* n* =* 184 (49%)	p *value^a^*
Age, years, median (IQR)	25 (20–40)	27 (21–43)	24 (20–37)	**0.008**
Age, years, mean (SD)	31 (13)	32 (13)	29 (13)	NC
Male, *n* (%)	247 (65)	124 (64)	123 (67)	0.550
Previous mTBI, *n* (%)^b^	82 (22)	47 (24)	35 (19)	0.242
Cause of injury^c^				
Fall, *n* (%)	135 (36)	75 (39)	60 (33)	0.235
Violence, *n* (%)	65 (17)	27 (14)	38 (21)	0.078
Bicycle, *n* (%)	58 (16)	35 (18)	23 (13)	0.141
Sport, *n* (%)	54 (14)	24 (12)	30 (17)	0.264
MVA, *n* (%)	43 (12)	19 (10)	24 (13)	0.310
Struck object, *n* (%)	17 (5)	12 (6)	5 (3)	0.106
Other, *n* (%)	3 (1)	1 (1)	2 (1)	0.601^f^
GCS score, *n* (%)^d^				
15	277 (73)	149 (81)	128 (82)	0.881
14	57 (15)	30 (16)	27 (17)	0.822
13	5 (2)	4 (2)	1 (1)	0.269^f^
Observed LOC, *n* (%)^e^	173 (70)	92 (71)	81 (70)	0.872
PTA 1–24 h	107 (28)	59 (30)	48 (26)	0.351
CT performed, *n* (%)	299 (79)	162 (84)	137 (75)	**0.031**
CT intracranial lesions, *n* (%)^g^	22 (7)	13 (8)	9 (7)	0.631
Level of care, *n* (%)				
Discharged home from ED	260 (69)	134 (69)	126 (69)	0.901
Observation <24 h	61 (16)	31 (16)	30 (16)	0.932
Neurosurgical admission	39 (10)	20 (10)	19 (10)	0.996
Another admission	18 (5)	9 (5)	9 (5)	0.908

^a^ *P* value for comparison between patients in the MRI study and patients with basic follow-up. Significant *p* values are shown in boldface. ^b^*n* = 374. ^c^*n* = 375. ^d^*n* = 339. ^e^*n* = 246. ^f^Exact unconditional test. ^g^*n* = 299, only patients with CT.

MRI, magnetic resonance imaging; IQR, interquartile range; SD, standard deviation; NC, not calculated; mTBI, mild traumatic brain injury; MVA, motor vehicle accidents; GCS, Glasgow Coma Scale; LOC, loss of consciousness; PTA, post-traumatic amnesia; CT, computed tomography; ED, emergency department.

**Table 2.** Patients Characteristics: MRI Study and Community Controls

*Variable*	*MRI study n* = 194	*Community controls* n* =* 78	p *value^a^*
Age, years, median (IQR)	27 (21–43)	28 (23–43	0.616
Male, *n* (%)	124 (64)	49 (63)	0.865
Previous mTBI, *n* (%)^b^	47 (24)	7 (9)	**0.004**
Education, years, median (IQR)^b^	13 (12–16)	13 (12–16)	0.407
Non-traumatic WMH	108 (56)	42 (54)	0.784
PCS at 3 months^c^	37/183 (20)	1/69 (1.4)	0.001^e^
PCS at 12 months^d^	25/167 (15)	1/66 (1.5)	0.005^e^
CT, hours, median (IQR, range)^f^	2.3 (1,6-3.9)		
Hours to MRI 72 h, median (IQR)^g^	58 (40–64)		
Days to MRI 3 months, median (IQR)^h^	94 (91–99)	95 (91–105)	0.088
Days to MRI 12 months, median (IQR)^i^	368 (364–374)	367 (361–375)	0.457

^a^ Significant *p* values marked show in boldface.^b^*n* = 271. ^c^PCS assessment in 183/194 (94%) of the patients and in 69/78 (88%) of the controls at 3 months. ^d^PCS assessment in 167/194 (86%) of the patients and in 66/78 (85%) of the controls at 12 months. ^e^Exact unconditional test. ^f^CT performed as part of the initial clinical assessment if deemed clinically indicated (*n* = 162). ^g^*n* = 193 ^h^MRI scans were performed in 152/194 (78%) of patients and in 73/78 (94%) of controls at 3 months. ^i^MRI scans were performed in 152/194 (78%) of patients and in 64/78 (82%) of controls at 12 months.

MRI, magnetic resonance imaging; IQR, interquartile range; mTBI, mild traumatic brain injury; WMH, non-traumatic white matter hyperintensities; PCS, post-concussion symptoms; CT, computed tomography.

In total, 44 patients had been recruited from the level 1 trauma center and 150 patients had been recruited from the municipal emergency clinic. The rates of subsequent hospitalization were 93% and 13%, respectively. Among patients recruited from the level 1 trauma center, more were injured in motor vehicle accidents (MVA) (30% vs. 4%, *p* < 0.001), than were patients recruited from the municipal emergency clinic, whereas among patients recruited from the municipal emergency clinic, more were injured as a result of violence (2% vs. 17%, *p* = 0.01) and more had GCS score 15 (70% vs. 85%, *p* = 0.03). There were no differences regarding age and sex in the two groups. Timing of imaging for patients and controls is presented in Table 2. MRI was performed at a mean of 52 h post-injury and 91% were examined within 72 h. MRI examinations were performed at all time points in 148 patients (76%) (Fig. 1). The SWI sequence was missing from the very early scanning in four patients. In the controls, FLAIR and SWI scan were missing from the 3 month scanning in one person.

In addition to the WMHs described separately, 32 patients and 4 controls had incidental non-traumatic findings such as cysts, angiomas, perivascular spaces, and calcifications.

### Intracranial traumatic lesions on CT and very early MRI

CT showed traumatic lesions in 8% (*n* = 13/162), and all these patients had lesions on very early MRI. Very early MRI showed traumatic lesions in 12% (*n* = 23/194) of the patients (Table 3). TAI was found in 6% (*n* = 11) and was the only finding in 4% (*n* = 7). Of the 32 patients where CT had not been deemed indicated, two had TAI lesions on MRI. All patients with TAI had lesions in the frontal lobe, one patient had a lesion in the temporal lobe, and one had lesions also in the corpus callosum. All 11 patients with TAI, as defined by lesions depicted on FLAIR, DWI and/or SWI in the pre-defined locations, had lesions on SWI, whereas 7 of the 11 had TAI lesions on FLAIR and 3 had lesions on DWI (Table 3). None had TAI depicted *only* on FLAIR or DWI. In the 13 patients with contusions, the lesions were visible on SWI in all 10 who had a valid SWI scan. In 12 of the 13, lesions were visible on FLAIR and in 8 were visible on DWI (Table 3). Among the controls, only two had a single hypointense lesion on SWI that was hyperintense also on FLAIR. These had no hypertension and had never sustained an mTBI.

**Table 3.** Traumatic Lesions on Very Early Mri by Sequences

	*All lesions*	*TAI*	*Contusions*	*EDH*	*SDH*	*tSAH*
Patients, n (%)	23 (12)	11 (6)^a^	13 (7)^b^	4 (2)^c^	3 (2)^d^	3 (2)
SWI^e^		11	10	3	3	2
FLAIR		7	12	4	3	3
DWI^f^		3	8	Not relevant		

^a^ Only TAI in 7 patients. ^b^Only contusions in 3 patients. ^c^Only EDH in 1 patient. ^d^Only SDH in 2 patients. ^e^SWI scan missing in 2 patients and unreadable in 1 patient. ^f^DWI scan missing in 1 patient.

MRI, magnetic resonance imaging; TAI, traumatic axonal injury; EDH, epidural hematoma; SDH, subdural hematoma; tSAH, traumatic subarachoid hemorrhages; SWI, susceptibility weighted imaging; FLAIR, fluid-attenuated inversion recovery; DWI, diffusion weighed imaging.

Out of patients without traumatic findings on CT, 5% (*n* = 8) had traumatic lesions on MRI, of which five had isolated TAI and three had combined TAI and contusions.

The patients who had been recruited from the trauma center had a higher frequency of traumatic findings on MRI than the patients who had been recruited from the municipal emergency clinic (25% vs. 8%, *p* = 0.002).

Patients with traumatic lesions on MRI were more often admitted to the neurosurgical department (52% vs. 5%, *p* = 0.001) than patients without lesions, and more often had PTA 1–24 h (52% vs. 28%, *p* = 0.016) (Table 4). In a follow-up analysis in patients with MRI findings, we subsequently explored if longer duration of PTA might be associated with lesions in the temporal lobe. We found that temporal lobe lesions were of all types (i.e., TAI, contusions, and hematomas) and were more common when PTA was 1–24 h (58% vs. 18%). The association was not statistically significant (OR 6.3 [CI: 0.93–42.7], *p =* 0.058).

**Table 4.** Characteristics of Patients with and without Traumatic Findings on MRI

	*MRI study*		
*Variable*	*Lesions on MRI* n* = 23*	*Normal MRI* n* = 171*	p *value^a^*
Age, median (IQR)	24 (21–38)	27 (21–44)	0.814
Age, mean (SD)	31 (12)	32 (13)	NC
Male, *n* (%)	16 (70)	108 (63)	0.548
Previous mTBI, *n* (%)^b^	5 (22)	42 (25)	0.756
Missing	0	1 (1)	NC
Cause of injury			
Fall, *n* (%)	10 (44)	65 (38)	0.613
Violence, *n* (%)	4 (17)	23 (14)	0.630^c^
Bicycle, *n* (%)	2 (9)	33 (19)	0.227^c^
Sport, *n* (%)	1 (4)	23 (14)	0.245^c^
MVA, *n* (%)	4 (17)	15 (9)	0.213^c^
Struck by object, *n* (%)	0	12 (7)	0.206^c^
Other, *n* (%)	1 (4)	0	NC
Unknown, *n* (%)	1 (4)	0	NC
GCS score, *n* (%)			
15	17 (74)	132 (77)	0.726
14	6 (26)	24 (14)	0.601^c^
13	0	4 (2)	0.601^c^
Unknown^d^	0	11 (6)	0.244^c^
LOC (%)			
Yes, *n* (%)n	11 (48)	81 (47)	0.967
No, *n* (%)	3 (13)	31 (18)	0.562^c^
Unknown, *n* (%)	9 (39)	59 (35)	0.662
PTA, 1–24 h (%)	12 (52)	47 (28)	**0.016**
Head CT findings, *n* (%)	13 (57)	0	NC
WMH, n (%)	12 (52)	96 (56)	0.719
Hours to MRI 72 h, median (IQR)^b^	58 (46–63)	57 (38–65)	0.672
Days to MRI 3 months, median (IQR)^d^	95 (91–100)	94 (91–99)	0.990
Days to MRI 12 months, median (IQR)^e^	369 (363–376)	367 (364–373)	0.911
Level of care, *n* (%)			
Discharged home from ED	5 (22)	129 (75)	**0.001**
Observation <24 h	5 (22)	26 (15)	0.436^c^
Neurosurgical admission	12 (52)	8 (5)	**0.001^c^**
Another admission	1 (4)	8 (5)	0.973^c^
PCS at 3-month^f^, n/total n (%)	7/21 (33)	30/162 (19)	0.119^c^
PCS at 12-month^g^, n/total n (%)	4/19 (21)	21/148 (14)	0.500^c^

^a^ Significant *p* values shown in boldface. ^b^*n* = 193. ^c^Exact unconditional test. ^d^MRI scans were performed in 165/194 (85%) of patients at 3 months. ^e^MRI scans were performed in 152/194 (78%) of patients at 12 months. ^f^PCS assessment in total 183 (94%) of patients at 3 months post-injury. ^g^PCS assessment in total 166 (86%) patients at 12 months post-injury.

MRI, magnetic resonance imaging; IQR, interquartile range; SD, standard deviation; NC, not calculated; mTBI, mild traumatic brain injury; MVA, motor vehicle accidents; GCS, Glasgow Coma Scale; LOC, loss of consciousness; PTA, post-traumatic amnesia; CT, computer tomography; WMH, non-traumatic white matter hyperintensities; ED, emergency department; PCS, post-concussion symptoms.

### Evolution of TAI over time

**FIG. 3.**
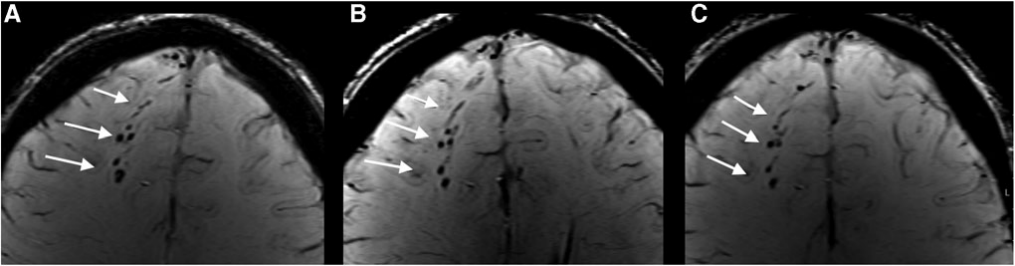
**(A–C)** Traumatic axonal injury (TAI) on susceptibility weighted imaging (SWI) scan in the transverse plane of the same patient as in Figure 4, acquired at three different time points. **(A)** Microbleeds (arrows) at the gray-white matter junction in the right frontal lobe on the very early SWI scan. The microbleeds persisted at the **(B)** 3 month and the **(C)** 12 month examination.

**FIG. 4.**
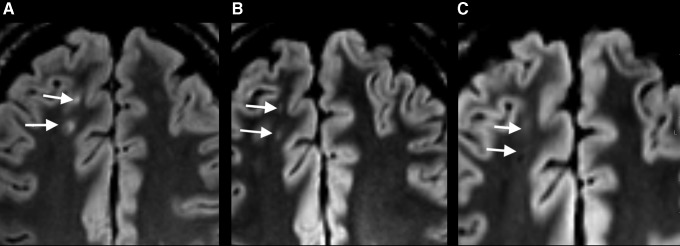
**(A–C)** Traumatic axonal injury (TAI) on fluid attenuated inversion recovery (FLAIR) scan in the transverse plane of the same patient as in Figure 3, acquired at three different time points. **(A)** Hyperintense lesions (arrows) at the gray-white matter junction in the right frontal lobe on the very early FLAIR scan. **(B)** At 3 months, the lesions were less conspicuous. **(C)** At 12 months, the lesions appeared even more isointense with surrounding normal white matter tissue.

Out of the 11 patients with TAI on very early MRI, all returned for the 3 month and 10 returned for the 12 month scanning. Based on the SWI scans, TAI was also detected at 3 and 12 months in all 11 patients with TAI lesions on very early SWI. The microbleeds tended to be smaller and less uniformly hypointense at 3 and 12 months (Fig. 3). Based on the FLAIR scans, TAI was detected at 3 and 12 months in only three of the seven patients with TAI lesions on very early FLAIR (Fig. 4). In these three patients, the lesions on FLAIR were more isointense with surrounding normal white matter. Based on the DWI scans, the three patients with TAI lesions on very early DWI had no lesions on the 3 and 12 month MRI.

### Evolution of other lesions over time

Out of the 13 patients with contusions on very early MRI, 9 returned for the 3 month scanning and 8 returned for the 12 month scanning. On the SWI scans, contusions were detected at 3 and 12 months in all patients with contusions on very early MRI. Using the FLAIR scans, contusions were detected in five patients at 3 and 12 months. Hence contusions were no longer visible on FLAIR scans in three patients, and MRI appeared normal. The EDH and SDH were resolved at the 3 and 12 month scans, whereas tSAH was visible on SWI at 3 and 12 months in one patient.

### IRR

When assessing the presence or absence of traumatic findings, the two raters assigned the images to the same category in 89% (73/82) of the patients. The overall IRR was good, with a Cohen's κ of 0.69 (95% CI 0.51–0.87, *p* < 0.001). When assessing the presence or absence of TAI, the raters assigned the images to the same category in 92% (75/82) of the patients. The IRR for TAI was moderate, with a Cohen's κ of 0.58 (95% CI 0.31–0.86, *p* < 0.001).

### Non-traumatic white matter hyperintensities

Non-traumatic WMHs were found in 108 (56%) of the patients with MRI and in 42 (54%) of the controls (*p* = 0.784). The frequency of WMH was not significantly different between patients with and patients without traumatic lesions on MRI (52% vs. 56%, *p* = 0.719) (Table 4). We subsequently performed a follow-up examination to explore if demographic and injury-related characteristics were associated with WMH, by dichotomizing the patients with mTBI without any traumatic findings on MRI (*n* = 171) into those with and those without presence of WMH (yes or no) and compared these two groups. Those with WMH (*n* = 96) were older than those without (*n* = 75) (38 [IQR = 23–51) vs. 23 [IQR = 20–32] years, *p* = 0.001), and there were more women among patients with WMH than among those without (56% vs. 72%, *p* = 0.034). No significant difference was found regarding frequency of previous TBI (*p* = 0.646), having PTA 1–24 h (*p* = 0.832) or MVA being the cause of injury (*p* = 0.819).

### Outcome

Follow-up rates were 94% (*n* = 183) at 3 months and 86% (*n* = 166) at 12 months. At 3 months, 21% of the patients with mTBI, but only 1.4% of the controls, met the criteria for PCS (Table 2). At 12 months, 15% of the patients and 1.5% of the controls met the criteria. At 3 months, 33% (*n* = 7) of the patients with, and 19% (*n* = 30) of the patients without traumatic MRI findings met the criteria for PCS (OR 1.96 [CI: 0.60–6.36], *p* = 0.119) (Table 4). At 12 months, the frequency of PCS was 21% (*n* = 4) and 14% (*n* = 21) (OR 1.16 [CI: 0.30–4.45], *p* = 0.500). When examining the patients with PCS at 3 months, MRI depicted traumatic lesions in 19% of these patients and in 10% of the patients without PCS. At 12 months, 16% of patients with PCS and 11% of patients without PCS had traumatic MRI findings.

## Discussion

To date, this is the largest prospective cohort study with longitudinal clinical MRI in patients with mTBI. Notably, a control group was scanned at the same three time points. Traumatic lesions were present in 12% on very early MRI. In 5% of the patients with negative CT, lesions were present on MRI, mainly because of the lack of TAI detection. TAI, contusion, and tSAH on SWI scans persisted for 12 months. There was no statistically significant difference in occurrence of PCS at 3 and 12 months between those with and those without traumatic findings or the occurrence of traumatic findings in patients with PCS at 3 and 12 months.

In the present study, we found similar or lower frequencies of traumatic findings than previous studies both for lesions on CT (5–39%)^3–7^ and on MRI (6–75%) in mTBI.^8–11^ The study population characteristics are important when comparing results among studies. Patients in the present study were recruited from a general practitioner-run outpatient clinic in addition to the ED at a level 1 trauma center, which also serves as a general hospital. All patients with mTBI in the catchment area present to these EDs if they seek acute medical assessment. Hence, we also recruited patients with less severe mTBI into the Trondheim mTBI follow-up study. In contrast, the Transforming Research and Clinical Knowledge (TRACK)-TBI study, a highly cited MRI study of 135 patients with mTBI, observed traumatic lesions on MRI in 43% of patients with mTBI triaged to acute head CT from EDs at three level 1 trauma centers in the United States.^10^ Notably, also had traumatic lesions on MRI 27% of the patients with normal admission CT, even if the distribution of GCS score was quite similar as in our study.^10^ The TRACK-TBI study, and also other studies recruiting patients only from academic trauma centers, report more MVA than the present study. Their results might reflect that their samples were skewed toward the more severe spectrum of mTBI or comprised more patients with multiple trauma.^9,10,30,31^ Interestingly, we observe a higher frequency of intracranial findings on MRI in the patients who had been recruited from the level 1 trauma center in this study. Hence, results from studies conducted in trauma centers may not apply to the most common patients with mTBI treated by general practitioners.

Moreover, in the present study, a relatively high proportion of the screened patients with mTBI were subsequently enrolled in the Trondheim mTBI follow-up study.^19^ The patients included in the MRI study were similar to the patients receiving basic follow-up on all variables except age, possibly because fewer teenagers were willing to participate in such comprehensive data collection. We consider that a representative selection among potential participants is crucial, yet such background information is seldom reported.

In the current study, the MRI was performed very early with 3 Tesla with a comprehensive imaging protocol including high resolution SWI, to increase sensitivity to microbleeds. Still, the frequency of traumatic lesions was low. It should also be noted that a low threshold for referrals to CT and MRI, which was the case in this study, can lead to a high frequency of normal imaging results.^4,32^ The low frequency of pathological findings in patients with normal CT, however, does not call for routine use of MRI in the acute phase in patients with typical mTBI; that is, patients who present to general hospitals and primary health care clinics.

### TAI

TAI was present in 6% of the patients with mTBI, but less frequent than the 13–28% reported in previous mTBI studies.^10,11,14,33,34^ As expected in a sample of mTBI, no lesions suspicious for TAI, such as point hemorrhages, were detected on CT. Only one patient (with GCS score 14) had TAI also in the corpus callosum, whereas no patients had TAI lesions in the basal ganglia, thalamus, or brainstem. As expected in this mTBI cohort, TAI lesions in clinical MRI were mainly located in the lobar white matter (Stage 1), because TAI lesions in deeper locations are associated with lower levels of consciousness, and hence more severe TBI grade.^35^

Importantly, SWI depicted more TAI than did FLAIR and DWI scans. Others have also found that SWI detects more TAI lesions than FLAIR.^36,37^ Hence, microbleeds in the lobar white matter are the main finding indicating TAI in mTBI, and SWI should be included in the mTBI scan protocols in addition to FLAIR and DWI.

### Evolution of TAI over time

The current study is the first longitudinal study of a cohort of patients with mTBI scanned at the same time points for 1 year. TAI could be detected on SWI during the entire year, but the lesions were less conspicuous 3 and 12 months after the injury. This finding is in line with previous longitudinal studies of patients with moderate and severe TBI using T2*gradient recalled echo (GRE) sequences.^38,39^ In contrast, some TAI lesions on FLAIR scans disappeared during the first 3 months after injury, as also observed in moderate and severe TBI.^39,40^ In summary, our findings indicate that in mTBI, the clinical MRI examinations can reliably be performed at a later time point. This might not be the case for all imaging techniques such as DTI and arterial spin labeling perfusion, which may be more sensitive to time since injury.^41^

### IRR

The agreement between the two raters was high, as they categorized the patients similarly in 9 out of 10 cases, which indicates that the clinical reading of MRI in mTBI can be sufficiently accurate, given that detailed criteria for the reporting are agreed upon. Despite a high agreement, the κ values were only moderate to good, likely explained by the large difference in prevalence in each category (e.g., TAI vs. no TAI), the so-called κ paradox.^42^ Previous studies have reported variable agreement, especially for TAI lesions, supporting our results.^13,36,39,43^

### Non-traumatic WMH

Although difficult, we attempted to differentiate TAI lesions from non-traumatic WMH on FLAIR scans. WMH were defined as hyperintense lesions on FLAIR, without hypointense lesions on SWI, which persisted unchanged across all time points on MRI. First, in line with another study,^44^ we found no difference in the frequency of non-traumatic WMH between the mTBI group and the controls, as expected based on these lesions considered not to be of traumatic origin.

In contrast, another study found that both hyperintense lesions on FLAIR and microbleeds on SWI occurred more frequently among patients than among controls.^45^ However, that study has been criticized for being difficult to interpret,^46^ because field strength and sequence parameters differed between patients and controls. Second, we found that patients with non-traumatic WMH were older, and more often female, than patients without non-traumatic WMH, which is consistent with findings in studies of WMH in the general population.^43,47–49^ Finally, we did not find any association between WMH and previous TBI or indicators of high-energy injuries, supporting that the findings classified as WMH in this study were not misclassified TAI lesions, but rather were unspecific WMHs. Nevertheless, we consider the high frequency of WMH to represent a potential source of error in the interpretation of the FLAIR sequence for TAI evaluation, especially if SWI or T2* are not performed. Microbleeds, on the other hand, were rare in the control group here, and were more likely to represent a traumatic finding when depicted on SWI in patients with mTBI in this age group. Because SWI also outperformed FLAIR in the detection of TAI, SWI should be included in an mTBI MRI protocol.

### MRI lesions related to clinical characteristics and outcome

The PTA duration was longer in patients with traumatic lesions, in line with previous studies.^15,50^ Our study also indicted that patients with long PTA duration more often have temporal lobe lesions. The pathophysiology of PTA is not fully understood, but the presented results might support the findings in a previous study, in which a disconnection between the medial temporal lobe and the default mode network was proposed in patients with PTA.^51^

Patients with traumatic MRI lesions had higher frequency of PCS at 3 months than patients without lesions, with an OR of 1.96. The difference was, however, not statistically significant. Previous studies of the association between traumatic findings on MRI and outcome report conflicting results, in studies of PCS^3,8,9,13,15,17^ and functional outcome,^10,13,17^ in studies applying univariable analyses,^3,8,13,15,17^ and in studies in which MRI findings have been included in multivariable analyses for outcome prediction.^9,10,17^ Although some report that traumatic lesions on clinical MRI were related to worse outcome after mTBI,^10,13^ others found no association.^3,8,9,13,15,17^ Based on our results, studies recruiting patients from only level 1 trauma centers might find a stronger association.^10^ Nevertheless, most studies analyzing the relationship between MRI findings and outcome in terms of PCS find no association. Still, the lack of association in the present study could represent a type 2 error. Given that PCS has the incidence shown in this study, approximately twice as many mTBI cases would need to be included to demonstrate an effect of traumatic findings on MRI on PCS frequency with an α of 0.05 and a β of 0.20. Hence, very large MRI studies are needed to evaluate the impact of traumatic MRI findings on outcome in mTBI samples in which most have normal brain imaging results. A range of biopsychosocial factors probably contributes to the self-reported problems after mTBI.^52,53^ Still, we find it plausible that damage to brain tissue as evidenced by clinical MRI plays a role in the complex pathogenesis of long-lasting PCS. Nevertheless, we consider that results from this and other studies do not support routine use of very early clinical MRI for prediction of PCS in patients who present acutely with mTBI.

### Strengths and limitations

One strength of this study was the prospective design. Further, we screened all eligible patients, used few exclusion criteria, and recruited patients from the only two healthcare facilities available for patients in the area with acute rain injuries, representing both hospital and primary care EDs. The follow-up rates for MRI scans was 85% at 3 months and 78% at 12 months, and the results for outcome evaluation were 94% at 3 months and 86% at 12 months post-injury, which was scientifically adequate.^54^ Indeed, follow-up rates were high in this mTBI population.^55^ Hence, we believe that the study population is representative of the wide spectrum of mixed-mechanism mTBI. Another strength of the study was the longitudinal design, with three MRI examinations at uniform time points both in patients and matched controls. Additionally, we had a state-of the art MRI protocol, which was the same in all patients and controls and was unchanged across all examinations. The inclusion of a control group, matched for key demographic characteristics, was a strength of this study. The control group was, however, intended to represent persons with fairly good brain health, and there might be underlying differences in the health of patients and community controls. For example, previous mTBI was more common among patients than among controls in this study. A tendency to sustain recurrent injuries in patients with TBI has also previously been reported,^56^ possibly reflecting that some lifestyle factors or concurrent health problems can increase the risk of TBI.

Although the included number of patients in the present study was relatively high, the low frequency of patients with traumatic findings led to a higher risk of a type 2 error in the analysis of the relation between MRI lesions and PCS. Inevitably, the estimates of the frequency of CT and MRI findings were also uncertain, because of the low number of findings.

Finally, our conclusions may not be valid for patients with mTBI who are >60 years of age . The upper age limit was chosen to reduce the burden of non-traumatic WMH on MRI, which increases with age.^48,49,57^ Especially in the later DTI analyses in the Trondheim mTBI follow-up study, a high load of WMH among participants could be a source of error.

## Conclusion

In this representative study of patients who sought medical evaluation after mTBI in both the primary care and the hospital setting, 12% had traumatic lesions on very early MRI. TAI was present in 6%, and was not detected by CT. Signs of TAI were best depicted by SWI and persisted in all cases up to the 12 month time point. We did not find any statistically significant relationship between traumatic findings detected by MRI and PCS, possibly because of the lack of power. Because the likelihood of traumatic findings is low, and the clinical significance of findings is unclear, our results do not support routine use of very early clinical MRI in mTBI. Moreover, because MRI depicted traumatic lesions by SWI for an extended time period after the trauma, the MRI examination can reliably be performed later as part of medicolegal evaluations or differential diagnosis in patients with persistent symptoms after mTBI.
